# Transfers to psychiatry through the consultation-liaison psychiatry service: 11 years of experience

**DOI:** 10.1186/1744-859X-7-10

**Published:** 2008-08-14

**Authors:** Christos Christodoulou, Katerina Fineti, Athanasios Douzenis, George Moussas, Ioannis Michopoulos, Lefteris Lykouras

**Affiliations:** 1 Second Department of Psychiatry, University of Athens Medical School, 'Attikon' General Hospital, 1 Rimini Street, 124 62, Athens, Greece; 2 Department of Psychiatry, General Hospital of Athens 'G. Gennimatas', 154 Mesogion Avenue, 115 27, Athens, Greece

## Abstract

**Background:**

There are only a few reports on issues related to patient transfer from medical and surgical departments to the psychiatric ward by the consultation-liaison psychiatry service, although it is a common practice. Here, we present a study assessing the factors that influence such transfers.

**Method:**

We examined the demographic and clinical backgrounds of a group of patients transferred from internal medicine and surgery to the psychiatric ward over an 11-year period. A comparison was made of this data with data obtained from a group of non-transferred patients, also seen by the same consultation-liaison psychiatry service.

**Results:**

According to our findings, the typical transferred patient, either female or male, is single, divorced or widowed, lives alone, belongs to a lower socioeconomic class, presents initially with (on the whole) a disturbed and disruptive behaviour, has had a recent suicide attempt with persistent suicidal ideas, suffers from a mood disorder (mainly depressive and dysthymic disorders), has a prior psychiatric history as well as a prior psychiatric inpatient treatment, and a positive diagnosis on axis II of the five axis system used for mental health diagnosis.

**Conclusion:**

The transfer of a patient to the psychiatric ward is a decision depending on multiple factors. Medical diagnoses do not seem to play a major role in the transfer to the psychiatric ward. From the psychiatric diagnosis, depressive and dysthymic disorders are the most common in the transferred population, whilst the transfer is influenced by social factors regarding the patient, the patient's behaviour, the conditions in the ward she/he is treated in and any recent occurrence(s) that increase the anxiety of the staff.

## Background

The department of psychiatry in a general hospital setting has a multidimensional role, providing inpatient care, maintaining strong interaction with community psychiatric services and offering specialist services to the general hospital wards either as part of the multidisciplinary approach to patient management or by offering specialist inpatient care to patients already hospitalised in other departments by transferring certain patients to the psychiatry department [1,2].

The consultation-liaison psychiatry service is the link between any general hospital ward and the department of psychiatry [3]. But what are the reasons for transferring a patient from a non-psychiatric bed to an inpatient psychiatric unit?

To the best of our knowledge, there are only a few reports on patient transfer issues although it is a common practice. In this context, in the present work we put forward our experience and thoughts on the factors that drive the patients transfer from general medicine to psychiatry.

We examined demographic and clinical backgrounds of a group of patients transferred from internal medicine or surgery to the psychiatric ward. A comparison was made of this data with data obtained from a group of non-transferred patients, also seen by the same consultation-liaison psychiatry service.

## Patients and methods

The present study was carried out at the Peripheral General Hospital of Athens 'G. Gennimatas', an approximately 650 bed community-based hospital with a 18 bed psychiatric unit that covers the greater Athens area. During the study period the psychiatric ward at 'G. Gennimatas' only received voluntary admissions, and operated as an open, short-term unit (the first author of this study worked at the above department during the study period).

The files of the patients transferred to the psychiatric unit by the consultation-liaison service between 1 March 1989 (opening of the inpatient psychiatric unit) and 31 December 1999 were reviewed. In the year 2000 the law for compulsory hospitalisation of the mentally ill in Greece changed, therefore, all the psychiatric units housed in general hospitals were obligated to also receive compulsory admissions. This change of status has influenced not only the atmosphere in the psychiatric unit but also the admissions by the consultation-liaison service.

The data collected from the review of the transferred patients' charts included: age, sex, marital status, ward from which the patient was transferred, current psychiatric complaint, medical diagnosis, length of hospital stay, prior psychiatric history, psychiatric inpatient treatment, psychiatric diagnosis and use of psychotropic medication; socioeconomic status was also deduced using the patients' files. This data was compared with data from non-transferred patients' files (control group, corrected for age and sex) during the year 1994–1995 (during this year the first author of this study was responsible for the consultation-liaison service).

The psychiatric diagnoses are according to the Diagnostic and Statistical Manual of Mental Disorders (DSM)-IIIR [4] and DSM-IV [5] categories. For quantitative comparisons a t test was employed, whereas for qualitative comparisons we used a two-tailed Fisher's exact test.

## Results

In total, 294 patients (139 men and 155 women) were transferred to the psychiatric ward during the 11-year period of the study (1989 to 1999). The mean number of transfers per year was 26.7, ranging from 18 (1989) to 35 (1991 and 1998). During the above time period the psychiatric unit offered inpatient treatment to 2,974 patients; thus, the admissions by the consultation-liaison service accounted for 9.9% of the total admissions. In the same period, the overall number of referrals for psychiatric assessment was 5,567; thus, 5.2% of the patients seen by the consultation-liaison service were eventually transferred to the psychiatric ward.

The control group consisted of 225 patients (110 men and 115 women). The majority of the control group came from medicine (156, corresponding to 69.3%), and the remainder (69, 30.7%) came from surgery; the majority of the transferred patients also came from medicine (215, 73.1%) and the remainder (79, 26.8%) from surgery.

Table 1 shows demographic data from the transferred and the control groups. There were no significant differences regarding age and sex between the two groups. The transferred group patients were more likely to be single, divorced, or widowed compared to the non-transferred group patients, who were more likely to be married. In all, 44 (15.6%) patients of the transferred group had serious social, family and financial problems versus 11 (4.8%) of the non-transferred group (Fisher's exact test, p < 0.001).

**Table 1:** Demographic data

	**Transfers**		**Control group non-transfers**		**Two-tailed Fisher's exact test (p value)**
	**(n = 294)**	**%**	**(n = 225)**	**%**	
**Marital status:**					
Married	92	31.3	130	57.8	< 0.001
Single	122	41.5	68	30.2	< 0.01
Divorced	35	11.9	9	4.0	< 0.01
Widowed	45	15.7	18	8.0	< 0.01
**Sex:**					
Male	139	47.2	110	48.9	NS
Female	155	52.8	115	51.1	NS
					
**Age **(t-test)	46.5 ± 17.3 (16–87)		49.2 ± 19. (14–85)		t = -1.65, NS

NS, not significant.

Among the 294 transferred patients, 223 (75.8%) had a prior psychiatric history whereas 71 (24.1%) did not. Of the non-transferred group, 142 (63.1%) patients had a previous psychiatric history whereas 83 (36.9%) did not. This difference between the two groups is statistically significant (Fisher's exact test, p < 0.01). Of the transferred patients, 124 (42.1%) had prior psychiatric inpatient treatment, whereas 170 (57.8%) did not have any psychiatric treatment in their history, versus 21 (9.3%) and 204 (90.6%) of the control group (Fisher's exact test, p < 0.001).

Table 2 shows the main psychiatric complaints of both groups. Suicide attempts and disruptive behaviour/non-compliance were the most often encountered psychiatric complaints in the transferred group. Suicide attempts (146) represent 49.6% of transfers, 103 (70.5%) of them being related to drug overdose (self-poisoning), whereas 43 were not drug related.

**Table 2:** Psychiatric complaint

	**Transfers**		**Control group non-transfers**		**Two-tailed Fisher's exact test (p value)**
	**(n = 294)**	**%**	**(n = 225)**	**%**	
Attempted suicide	146	49.6	50	22.2	< 0.001
Psychiatric history/medication	23	7.8	41	18.2	< 0.001
Psychiatric symptomatology*	94	32.0	93	41.3	< 0.05
Disruptive behaviour/non-compliance	31	10.5	11	4.9	< 0.05
Subjective complaints without objective findings	0	0.0	12	5.3	< 0.001
Not described	0	0.0	18	8.0	< 0.001

*Including: confusion, agitation, depression, anxiety and delusions.

The mean hospital stay for the transferred patients was 26.31 ± 21.15 days (the hospital stay of 23 patients who left against medical advice is not included). During the 11-year period of the study, the longest mean hospital stay for the patients admitted through the outpatient psychiatric clinic and the emergency department (20.9 ± 22.4 days) was observed in 1995; nevertheless, the mean hospital stay for the transferred patients was significantly greater than the above number (t = 2.88, p < 0.01). We noticed that the patients with suicide attempts that were not drug-related (43) together with the patients with serious social problems (46) had the longest hospital stays (table 3).

**Table 3:** Average hospital stay (in psychiatric ward) in days

**Transfers to psychiatry ward by consultation-liaison (C-L) service**		**t Test**	**p Value**
Suicide attempts, not drug related (n = 43)	Remainders of the transferees (n = 228*)		
38.8 ± 26.3	25.2 ± 20.6	3.21	< 0.01
Serious socioeconomic problems (n = 46)	Remainders of the transferees (n = 225)		
40.2 ± 31.0	25.3 ± 21.3	3.11	< 0.01

*A total of 23 patients who left against medical advice are not included.

Table 4 shows the diagnoses and comparison of the two groups. The transferees were more likely to have been diagnosed with a mood disorder (including bipolar disorder types I and II, unipolar depression, dysthymic disorder) or a personality disorder, whereas the non-transferred were more likely to have been diagnosed with adjustment disorder as well as having 'no psychopathology'. In the other diagnostic categories there are no significant differences. In the transferred group, 23 patients had diagnoses on both axes I and II of the five axis system used for mental health diagnosis, compared with 9 non-transferred patients with the same pattern. Thus, overall, 56 of the transferees (19.0%) compared to 19 (8.4%) patients of the control group had a diagnosis on axis II (Fisher's exact test, p < 0.001). Of the transferred patients, 21 had a second diagnosis on axis I related to addictions, versus 13 patients of the control group. No diagnosis was made for 23 of the transferees and 16 of the non-transferred patients.

**Table 4:** Psychiatric diagnoses

	**Transfers**		**Control group non-transfers**	
	**(n = 294)**	**%**	**(n = 225)**	**%**
Delirium	33	11.2	36	16.0
Addictions	27	9.2	22	9.8
Schizophrenia	32	10.9	22	9.8
Other psychotic disorders	24	8.2	12	5.3
Mood disorders	78^a^	26.5	38^a^	16.9
Anxiety disorders	13	4.4	15	6.7
Somatoform disorder	7	2.3	11	4.9
Personality disorders	33^b^	11.2	10^b^	4.4
Adjustment disorders	18^b^	6.1	32^b^	14.2
Eating disorders	3	1.0	1	0.4
No psychopathology	3^a^	1.0	10^a^	4.4
Undiagnosed	23	7.8	16	7.1

Two-tailed Fisher's exact test:^a^p < 0.05, ^b^p < 0.01.

Table 5 shows the medical diagnoses for the two groups. We note that the number of injured/poisoned patients in the transferred group was significantly greater than the number of the corresponding non-transferred patients.

**Table 5:** Physical problems

	**Transfers (n = 294)**	**Control group (non-transfers; n = 225)**
Injuries/poisoning	146^a^	82^a^
Central nervous system diseases	35	19
Vascular diseases	17	16
Gastrointestinal tract diseases	18	25
Cancer	8	11
Endocrine diseases	16	10
Musculoskeletal diseases	10^b^	17^b^
Urinary tract diseases	5	1
Kidney diseases	6	2
Hematological diseases	5	1
Infectious diseases	8	6
Other diseases	11	15
Not clarified	9^a^	20^a^

Two-tailed Fisher's exact test: ^a^p < 0.01, ^b^p < 0.05.

## Discussion

During the study period (1989 to 1999), the transfers to the psychiatric unit handled by the consultation-liaison service accounted for approximately 9.9% of total admissions, with transfers representing the third source of admissions to the unit after the psychiatric emergency service (59.6%) and the psychiatric outpatient clinic (27.3%). The mean number of admissions per year was 26.7, or 5.3% of the referrals for psychiatric assessment during the 11-year period of the study. Similar percentages in the literature range from 8 to 14.9% [6-9].

In fact, the above numbers and percentages of transfers to psychiatric wards may seem relatively small given that psychiatrists reportedly believe that psychopathology in the hospitalised population at any moment, even with conservative estimations, exceeds 30% and ranges from 30 to 50% [10]. Psychiatric units have been said to be reluctant to receive patients transferred from the general hospital and this has been an important issue. Their 'preference' to patients with psychiatric diagnoses only is based not only on the pressure from the community for such admissions, but also on the argument that patients with somatic illnesses may exert a 'negative' influence on the therapeutic environment or are difficult to take care of [11,12].

At this point, we should perhaps clarify that the term 'difficult to take care of' usually refers to those patients who present with a variety of, mainly behavioural, problems in addition to their somatic illness, which actually makes them 'not wanted' in any ward [13-15]. Some of these problems may have been the reason that led their physicians to refer them for a psychiatric consultation or even discuss a transfer to psychiatry in the first place.

Marital status seems to be a basic discriminating factor between the two groups. Transferred patients were more likely to be single, divorced or widowed compared to controls that were more likely to be married. The same conclusion was reported by Leibenluft *et al*. [6]. The patient spouse and/or family seem to play an important role in the compliance to inpatient treatment in any general hospital ward and make the need for a transfer to psychiatry less likely [16-19].

Serious social (unemployment, extreme poverty, homelessness, lack of health insurance, etc) and family problems also seem to prevail in the transferred group. The absence of social support systems makes psychiatric inpatient treatment and the transfer to psychiatry more likely [18-23].

The transferred patients were significantly more likely to have a prior psychiatric history and a prior inpatient psychiatric treatment compared to the non-transferred group. It has been reported that the best predictors of hospitalisation are previous rehospitalisations, more severe symptoms and dissatisfaction with family relations [24]. However, a significant number of transferees (58.6%) had their first inpatient psychiatric treatment after their admission to the general hospital for the treatment of a physical illness, and this happens in the majority of the transferred patients. How can we explain this number? This is probably due to the relatively poor psychiatric care system in Greece. Some of the inpatients with suicide attempts (especially the ones with a first suicide attempt without prior psychiatric history), who would have been referred to an outpatient psychiatric service after leaving the hospital ward, need to remain for a few days in the psychiatric unit to ensure they have regained adequate control of their life. Another reason would be that psychiatric services are not readily available in general or not friendly enough to people who may need them at their time of need. Thus, a long-standing psychiatric problem is often revealed, or it is seen how important it is, or it is aggravated, when a patient is hospitalised for a medical problem. We would even go as far as to say that it seems the presence of a psychiatric unit in a general hospital setting makes psychiatry more available, or more 'justifiable', at least to people with coexisting medical problems.

The mean hospital stay of the transferees was longer than the mean hospital stay of the direct psychiatric admissions. The co-existence of medical or surgical problems together with psychiatric problems, for instance, a serious trauma after a suicide attempt, sometimes requires a long hospital stay and makes a longer hospital stay more likely in the transferees [25-27]. By contrast, medical co-morbidity was present in a substantial number of psychiatric inpatients in the general hospital units and this was associated with a prolonging of the length of their hospital stay as well [28]. The interaction of depression, which is the most common diagnosis amongst the transferred inpatients, and physical illness, has been reported to increase the length of stay in psychiatric units [29]. Nevertheless, in our study there were no important differences in the presence of physical illness between the two groups, excepting traumatic injuries and self-poisoning after attempted suicide, more often found in the transferred group.

The social conditions (marital status, unemployment, extreme poverty, homelessness, lack of health insurance etc) that some of the transferees were experiencing can give an additional explanation for the longer stay in the psychiatric ward [30,31].

The majority of the transferees had a recent suicide attempt (46.6%). This percentage proved to be higher compared to the 19–40% reported by similar international studies [6,7]. Suicide attempts are reported to be increasing in many countries. Consequently, attempted suicide is a regular reason for admission to a general hospital for both sexes [32-34].

Undoubtedly, a suicide attempt is among the conditions that alarm and sensitise physicians on medical and surgical wards. A recent suicide attempt, or a suicide attempt that takes place within a hospital ward, alerts the physicians and makes them very sensitive to any thought or action that could be considered self-destructive, even months after the attempt. What is more, it is not unusual for patients with a recent suicide attempt or suicidal ideation or major depression to be treated in overcrowded wards or on high floors near windows that cannot be safely locked, or in rooms that cannot be easily inspected by the nursing station [7]. The transfer of such patients to the psychiatric unit is dictated not only by the above-described lack of rehabilitation psychiatric services but also by pressure from physicians and, of course, by the understanding on the psychiatrist's side of the stress the physicians and the staff involved in treating such patients go through.

Behavioural problems and non-compliance are often encountered in the transferred patients (12%). Although psychiatrists usually try to keep such patients in the medical and surgical wards, when the efforts of the physicians are aimed rather at controlling the patients' impulsivity and disruptive behaviour than on the treatment of their somatic illness, their transfer to psychiatry often appears the only way to deal with them. In addition, the negative feelings of the doctors, the staff, and the rest of the patients treated in the same wards towards such 'difficult' patients create a burden carried not only by the people around them but also by the patient [6,7,13-15].

As for the psychiatric diagnoses, the mood disorders (mainly depressive and dysthymic disorder) and the disorders on axis II seem to discriminate the two groups. Specifically, the transferred group was significantly more likely to have a mood disorder or a disorder on axis II. Depression is the most common diagnosis in patients suffering from a physical illness, and it was evaluated either by self-rated depression scales or by structured psychiatric interview [35-38]. However, at this point we would like to stress again that in the present study the difference in the diagnoses between the two groups is mainly attributed to the increased number of transferees with suicide attempts.

## Conclusion

According to our findings, the typical transferred patient, either female or male, is single, divorced or widowed, lives alone, belongs to a lower socioeconomic class, presents with a disturbed and disruptive behaviour, has a recent suicide attempt with persistent suicidal ideas, suffers from a mood disorder, has a prior psychiatric history and a diagnosis on axis II. Psychiatric diagnosis on axis I (except mood disorders) does not seem to play an important role in the decision of transferring a patient to the psychiatric ward. It is also worth mentioning that the medical diagnosis does not seem to play a major role in the transfer to the psychiatric ward. As for the future, it might be of help if our efforts aim at considering and testing in the long run, by prospective studies, reliable criteria and factors, that should be acknowledged every time a transfer to psychiatry is decided.

## Limitations

The above study of the factors that influence the transfer of inpatients from the medical and surgical wards to psychiatry has the limitations of any retrospective study. The socioeconomic status of the transferees was deduced from information from the patients' social history; such information included: lack of health insurance, lack of permanent residence or homelessness, prolonged unemployment, lack of any income. The severity of the psychiatric and the physical illness are not precisely assessed. During the study period the psychiatrists who were responsible for the patients' transfer presented here were not the same person.

## Competing interests

The authors declare that they have no competing interests.
